# Prediction of the Potential Distribution of Common Pochard (*Aythya ferina*) and Marbled Teal (*Marmaronetta angustirostris*) in Iran Under Future Climate Scenarios

**DOI:** 10.1002/ece3.73971

**Published:** 2026-07-06

**Authors:** Abbas Naqibzadeh, Ruhollah Asgari, Cezary Mitrus, Attila Németh, Salvatore Surdo

**Affiliations:** ^1^ Department of Nature Conservation, Zoology, and Game Management, Faculty of Agricultural and Food Sciences and Environmental Management University of Debrecen Debrecen Hungary; ^2^ Doctoral School of Animal Science University of Debrecen Debrecen Hungary; ^3^ Department of Environment Gandoman, Shahr‐e Kord Chaharmahal and Bakhtiari Province Iran; ^4^ Institute of Environmental Biology, Department of Vertebrate Ecology and Paleontology Wrocław University of Environmental and Life Sciences Wrocław Poland; ^5^ BirdLife Hungary–Hungarian Ornithological and Nature Conservation Society Budapest Hungary; ^6^ Department of Agricultural, Food, and Forestry Sciences University of Palermo Palermo Italy

**Keywords:** climate change, climate refugia, common pochard, marbled teal, species distribution modelling, wetland conservation

## Abstract

Climate change is reshaping ecological communities worldwide, altering species distributions, abundance, and habitat suitability. We modelled the potential distribution of two duck species, the Common Pochard ( *Aythya ferina* ) and the Marbled Teal ( *Marmaronetta angustirostris* ), across Iran under current and future climate scenarios. Despite differences in optimal model performance (MaxEnt for Common Pochard and GAM for Marbled Teal), both species exhibited a convergent response to climate change, with a projected shift toward high‐elevation montane wetlands under extreme scenarios. Both species showed: (1) loss of lowland habitats; (2) expansion into montane wetlands; (3) increasing suitability in the Alborz and Zagros ranges. Under the SSP5–8.5 scenario (2081–2100), representing an extreme climate pathway characterised by very high greenhouse gas emissions, both species exhibited substantial increases in climatically suitable habitat. The Marbled Teal showed an expansion of up to 3711 km^2^, while the suitable habitat of the Common Pochard increased to 21,671 km^2^, corresponding to gains of 167.6% and 137.3%, respectively. These results indicate that both species may possess comparable capacities to persist under future climatic conditions. However, the projected expansions appear to be strongly associated with the availability of high‐altitude aquatic ecosystems, highlighting the crucial role of montane wetlands as potential climate refugia. Consequently, conservation planning should incorporate projections of future habitat suitability, with particular emphasis on the protection, restoration, and sustainable management of mountain wetland habitats to support the long‐term persistence of both species under changing climatic conditions.

## Graphical abstract

Predicting waterbird habitat changes under future climate scenarios.

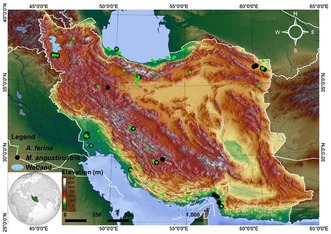

## 1 Introduction

Global biodiversity is undergoing an unprecedented decline, with extinction rates far exceeding natural background levels. This crisis, often referred to as the sixth mass extinction, is primarily driven by anthropogenic pressures such as habitat loss, overexploitation, invasive species, and climate change (Ceballos et al. 2015, 2017; Ceballos and Ehrlich 2023; Montràs‐Janer et al. 2024). Birds, due to their ecological sensitivity and well‐documented distributions, serve as valuable indicators of environmental change and are widely used in climate‐impact assessments (Huntley et al. 2006; Tingley et al. 2009; Potvin et al. 2016).

Climate change is expected to alter species distributions by reshaping temperature and precipitation regimes, modifying habitat suitability, and disrupting ecological interactions (Li et al. 2025; Song et al. 2026). Species' habitats are changing due to climate change; consequently, they are changing over time, becoming suitable, unsuitable, and even retaining suitability for the species. This phenomenon is known as climate refugia (Kavousi and Keppel 2017; Vaissi and Mohammadi 2024).

Climate refugia are habitats to which species can potentially migrate or expand as environmental conditions change. Climate refugia are classified into two types based on their geographical association: Ex‐situ refugia are located in areas currently abandoned by the species but are expected to be suitable under changing conditions, and in situ refugia, which means the current areas are suitable and inhabited by the species that are projected to remain suitable under climate change in the future (Kavousi and Keppel 2017; Baumgartner et al. 2018; Vaissi and Mohammadi 2024).

Wetlands are among the most valuable and biologically rich ecosystems globally, playing a fundamental role in sustaining biodiversity and providing a wide range of ecosystem services essential for human well‐being (Convention on Wetlands 2018). These ecosystems support numerous ecological processes and serve as critical habitats for a diverse array of species, particularly waterbirds, by providing breeding, nesting, foraging, wintering, and migratory stopover sites. In addition, wetlands contribute significantly to flood mitigation, water purification, groundwater replenishment, nutrient cycling, and carbon storage, thereby supporting both ecological integrity and climate regulation (Convention on Wetlands 2025). Owing to their exceptional ecological importance, wetlands are widely regarded as biodiversity hotspots and are central to global conservation efforts.

Despite their ecological and socioeconomic value, wetlands are experiencing unprecedented levels of degradation and loss worldwide. Historical assessments suggest that approximately 21%–35% of global wetland area has disappeared since the eighteenth century, while many remaining wetlands continue to be adversely affected by land‐use change, hydrological modifications, pollution, biological invasions, and unsustainable resource exploitation (Davidson 2014; Ramsar Convention Secretariat 2018). The impacts of climate change further exacerbate these pressures by altering hydrological regimes, increasing the frequency and severity of drought events, and reducing the spatial extent and ecological functioning of wetland ecosystems (Dudgeon et al. 2006; Pant et al. 2024; Bezzalla et al. 2025; Singh and Prakash 2025). As a consequence, the long‐term persistence of many wetland‐dependent species is increasingly at risk.

Among the taxa most closely linked to wetland ecosystems are waterbirds, which rely on these habitats throughout various stages of their life cycle and are widely recognised as effective indicators of ecosystem condition and environmental change (Ma et al. 2009). Nevertheless, numerous waterbird populations have experienced substantial declines in recent decades, primarily as a result of habitat loss, wetland degradation, climate change, and growing anthropogenic pressures on freshwater and coastal ecosystems (Rosenberg et al. 2019; https://www.wetlands.org). Consequently, understanding how future environmental changes may influence the distribution and availability of suitable wetland habitats is essential for developing effective conservation strategies and ensuring the long‐term persistence of wetland‐dependent bird species.

Understanding how species respond to climate change‐driven changes in habitat availability is therefore essential for effective conservation planning (Keppel et al. 2012; Trew and Maclean 2021).

The Marbled Teal ( *Marmaronetta angustirostris* ) is a globally threatened species, with an estimated population size of 10,000–42,000 (BirdLife International 2022). This species has been undergoing declines across much of its range in recent years. This is particularly true in the Middle East, where the species was once widespread as a breeder throughout much of the region. There is considerable uncertainty in the size and trends of the South West Asia population, which is primarily concentrated in the Mesopotamian marshes of Iran and Iraq (Aghababyan et al. 2025). These marshes are believed to have supported up to approximately 80% of the global population in the recent past. The presence of large flocks during the winter of 2010 is thought to have resulted from the congregation of individuals from various areas due to widespread drought, alongside the restoration of the Mesopotamian marshes (Salim 2010). The species breeds in temporary and unpredictable Mediterranean‐type wetlands (Green 2000, 2007) as well as in relatively dry, steppe‐like areas with shallow freshwater, brackish, or alkaline ponds that have a well‐vegetated shoreline and abundant emergent and submergent vegetation (Green 1993; Kear 2005; Sebastián‐González et al. 2013). Additionally, it may inhabit slow rivers and saline coastal lagoons, and man‐made wetlands, including fish‐rearing ponds, small reservoirs, and sewage farms (Green 1993). While it prefers brackish wetlands, it typically avoids waters with high salinity.

Common Pochard ( *Aythya ferina* ) is classified as a globally vulnerable species (BirdLife International 2021; Mischenko et al. 2020; Mischenko et al. 2022; Khan et al. 2025). The species breeds across a range that extends from Western Europe, through Central Asia, to South‐Central Siberia and Northern China. The Common Pochard has an estimated global population of 760,000–790,000 mature individuals (Wetlands International 2025). This species predominantly inhabits well‐vegetated freshwater ecosystems, particularly eutrophic to mesotrophic wetlands such as marshes, swamps, lakes, and slow‐moving rivers characterised by extensive open‐water areas and abundant emergent vegetation. During the breeding season, it also occupies a variety of saline, brackish, and soda lakes, and occasionally utilises sheltered coastal bays. This broad habitat use demonstrates the species' ecological flexibility and capacity to exploit diverse aquatic environments (Kear 2005).

Both species of the Common Pochard and Marbled Teal are present in Iran as wintering and breeding populations, though their status may vary locally depending on wetland conditions. Efforts to monitor and protect their habitats, as well as to raise awareness about the importance of wetland conservation, are essential for ensuring the survival of these species within the country. To understand their distribution patterns and effectively conserve them specifically within Iran, it is important to investigate the key environmental factors that influence them in this region.

Climate change poses a significant threat to global diversity, leading to increased temperatures, altered precipitation patterns, and a rise in extreme weather events. These changes impact the availability of suitable habitats both spatially and temporally (Hota et al. 2025). Species distribution models (SDMs) are computational tools that combine species occurrence data with environmental variables to assess the potential effects of climate change on species distribution patterns over time (Allouche et al. 2006; Cerasoli et al. 2021; Hota et al. 2025; Li et al. 2025; Shi et al. 2025). By implementing habitat modelling for species distributions under future climate scenarios, conservationists can gauge the influence of climate on species' realised niches, prioritise areas for conservation, identify potential climate refugia for protection, and explore options for assisted migration and connectivity (Cerasoli et al. 2021; Hota et al. 2025).

This study aims to: (1) identify key environmental factors influencing the distribution of Marbled Teal and Common Pochard in Iran; (2) map current areas of potential suitability; and (3) project future distributions under multiple climate scenarios to assess potential range shifts and conservation implications. The findings will help inform decisions regarding suitable areas for habitat conservation.

## 2 Materials and Methods

### 2.1 Study Area

**FIGURE 1:**
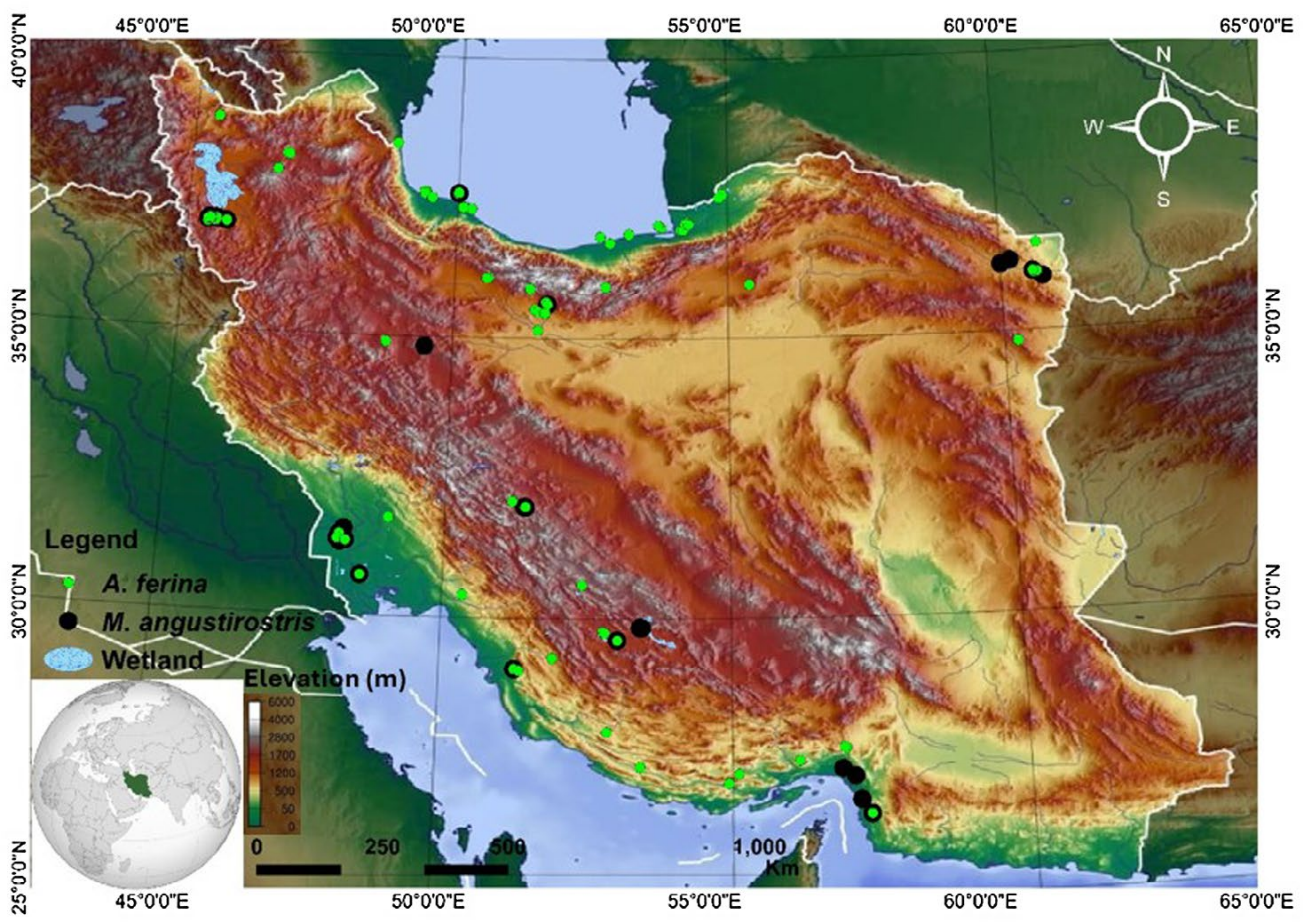
Map of Iran showing the study area with species occurrences and topographic background.

Iran's physiography is dominated by two major mountain systems (Figure 1): the Alborz Mountains in the north and the Zagros Mountains stretching from northwest to southeast. These mountain ranges enclose the extensive Central Iranian Plateau, which descends eastward into the low‐lying Lut and Kavir deserts. This elevational gradient creates a remarkable compression of climatic zones over short distances.

The country's complex topography drives pronounced climatic heterogeneity. The Alborz and Zagros ranges intercept moisture‐bearing systems, creating orographic precipitation gradients: annual rainfall exceeds 1500 mm on the northern slopes of the Alborz but drops below 50 mm in the central deserts. Temperature regimes vary correspondingly, with mean annual temperatures ranging from 9°C in montane areas to 22°C in lowland basins (Azad and Ahmadi 2024). Over 82% of Iran's territory is classified as arid or semi‐arid, yet mountain zones maintain more mesic conditions, supporting permanent rivers, springs, and wetlands that contrast sharply with surrounding drylands. This topographic complexity positions Iran as an ideal natural laboratory for investigating altitudinal climate refugia. Mountain wetlands, ranging from small alpine peatlands to larger montane lakes, represent persistent aquatic habitats that may buffer species against lowland aridification.

### 2.2 Species Occurrence Data

Occurrence data were compiled from both field surveys and publicly available biodiversity databases. Field surveys were conducted during the breeding seasons (May to August) from 2023 to 2025 and recorded species presence, active nests, geographic coordinates, and abundance. To complement these observations, additional occurrence records were obtained from the Global Biodiversity Information Facility (GBIF; https://www.gbif.org/).

In total, 275 occurrence records were collected for the Common Pochard and 260 for the Marbled Teal. Following data cleaning, which involved the removal of duplicate records and observations outside the breeding period, 128 presence records for the Common Pochard and 139 for the Marbled Teal were retained for species distribution modelling (Appendix 1, Supporting Information). Of these, approximately 105 records originated from field surveys, whereas 162 records were sourced from GBIF. The spatial distribution of all occurrence records used in the analyses is presented in Figure 1.

### 2.3 Environmental Variables

Selecting comprehensive and precise environmental variables is crucial for species distribution modelling. Habitat suitability models help us to project future distribution to understand species dispersion over time under changing their habitat. All spatial data processing, variable selection, and species distribution modelling were conducted in R version 4.5.2 (R Core Team 2025) using RStudio (Posit Team 2026). Current and future bioclimatic variables were downloaded directly within the R environment using the geodata package (Hijmans et al. 2024), which provides programmatic access to the WorldClim version 2.1 database (Fick and Hijmans 2017). Additional environmental layers (land use, ecoregions, topography, soil moisture) were processed using the terra package (Hijmans et al. 2024) for raster manipulation and the sf package (Pebesma 2018) for vector data operations. Variable correlation analysis and multicollinearity assessment to avoid large eigenvalues in the results were performed using the ‘corrplot’ (Wei and Simko 2021) and ‘usdm’ (Naimi et al. 2014) packages.

The habitat suitability modelling relies on several environmental variables. These include land use, ecoregion, and water resources (source: https://diva‐gis.org/index.html). Climatic data, composed of 19 bioclimatic variables for the current period (1970–2000), were obtained from the WorldClim version 2.1 database (http://www.worldclim.org/). Additionally, topographic information such as altitude (source: https://download.gebco.net/) and soil moisture data (source: https://nsidc.org/data/spl3smap/) were also utilised. To project future data for the period from 2021 to 2100, we incorporated four coupled scenarios based on Shared Socioeconomic Pathways and Representative Concentration Pathways (SSP‐RCPs). These scenarios align with the Coupled Model Intercomparison Project Phase 6 (CMIP6) and include SSP1‐RCP2.6, SSP2‐RCP4.5, SSP3‐RCP7.0, and SSP5‐RCP8.5 (Eyring et al. 2016). To assess the effects of future climate change on bird distribution, we sourced future climate data from the WorldClim version 2.1 database (https://www.worldclim.org/data/cmip6/cmip6_clim2.5m.html).

Environmental variables were processed using ArcGIS 10.8.2, where raster files were converted and masked to the study area. Climate variables were downloaded directly from the WorldClim website within the R environment and then prepared to match the extent and resolution of the other environmental variables.

To avoid multicollinearity issues and improve prediction accuracy, we removed highly correlated environmental variables. Specifically, for any pair of variables with a Pearson correlation coefficient |*r*| > 0.85, only one variable was retained.

We developed two distinct modelling approaches to project future suitable habitats, each using different sets of environmental variables. For both approaches, we generated ensemble maps weighted by model performance.

First, the statistical approach used all remaining environmental variables (after correlation filtering) plus climate scenarios. This approach aimed to quantify the contribution of each variable to species distribution patterns without a priori selection.

Second, the ecological approach focused on variables known to directly influence habitat quality and species requirements. In addition to the correlation filter (|*r*| < 0.85), we prioritised four key climate variables—Annual Mean Temperature (Bio 1), Max Temperature of Warmest Month (Bio 5), Annual Precipitation (Bio 12), and Precipitation of Driest Quarter (Bio 17)—based on their known ecological importance for the species (see Appendix 2 in Supporting Information). This approach aimed to show the extent to which suitable areas meet the species' biological needs.

In total, we retained 17 variables for the statistical approach and 26 variables for the ecological approach (Appendix 2 in Supporting Information).

To combine the two approaches into a single ensemble map, we weighted each approach by its predictive performance. We used the True Skill Statistic (TSS) for classification accuracy and the Boyce index for spatial reliability (suitable for presence‐only data). For each approach, we summed the TSS and Boyce values, then divided the weight of each approach by the total sum of weights. The final ensemble map was obtained by summing the product of each approach's map multiplied by its respective weight.

### 2.4 Species Distribution Modelling Algorithms

Species distribution models (SDMs) are one of the research hotspots in ecology, biology, and conservation (Wang et al. 2025), particularly with the increasing impact of climate change in recent years, which poses an unprecedented threat to global biodiversity (Sattar et al. 2021; Gaget et al. 2024; Delacruz and Numa 2024).

SDMs were calibrated using four algorithms implemented through the biomod2 framework (Thuiller et al. 2024), which provides a unified interface for multiple modelling techniques. The selected algorithms were (i) Generalised Linear Models (GLM), implemented via the glm function from the R base package stats (R Core Team 2025), with a binomial error distribution and logit link function; (ii) Generalised Additive Models (GAM), fitted using the mgcv package (Wood 2017), with smoothing splines applied to continuous predictors to capture non‐linear responses; (iii) Random Forest (RF), implemented through the randomForestSRC package (Ishwaran and Kogalur 2025), selected for its robustness to correlated predictors and ability to model complex interactions. The number of trees was set to 500, with default node size parameters. Finally, (iv) Maximum Entropy (MaxEnt), implemented using the maxnet package (Phillips et al. 2017), provides a regularised multinomial logistic regression approach as a modern R‐native alternative to the legacy Java‐based MaxEnt software. This implementation allows seamless integration within the R modelling workflow.

The performance of ensemble models was assessed using a multi‐metric approach to quantify variability among estimates. Evaluation metrics encompassed the True Skill Statistics (TSS) as a simple and intuitive measure for the performance of distribution models when predictions are expressed as a presence‐absence map (Allouche et al. 2006). The Area Under the Curve‐Receiver Operating Characteristics Statistics (AUC) curve provides an alternative technique for the assessment of the accuracy of ordinal score models. The construction of ROC curves uses all possible thresholds for classifying the scores into confusion matrices, obtaining each matrix's sensitivity and specificity; then comparing sensitivity against the corresponding proportion of false positives (Shabani et al. 2018), Boyce‐Code (BCNF), and Cohen's Kappa (KAPPA), which statistic is the most widely used measure for the performance of models generating presence–absence predictions.

All models were calibrated using 70% of the presence data for training and 30% for validation, with 10‐fold cross‐validation repeated three times to account for stochasticity in model fitting. We (1) extracted elevation values for all suitable cells with > 0.5 probability, (2) overlaid suitability maps with Iran Wetland Inventory data, and (3) compared elevation distributions between ‘All suitable areas’ and ‘Suitable areas’ overlapping existing wetlands.

### 2.5 Species Range Change

We used the BIOMOD_Projection function from the biomod2 package (Thuiller et al. 2024) to generate spatial projections of habitat suitability under current and future climate scenarios. Subsequently, we applied the BIOMOD_RangeSize function, which compares binary presence‐absence projections between time periods to quantify range dynamics. This function produces a table containing the absolute metrics Loss, Absent, Stable, and Gain, as well as the derived relative metrics Percent Loss, Percent Gain, and Range Change (Thuiller et al. 2024). Additionally, the function generates a categorical spatial map highlighting areas of habitat gain, loss, and stability. All range change analyses were performed within the biomod2 ensemble modelling framework.

## 3 Results

True Skill Statistics (TSS), Operating Characteristics Statistics (AUC), Boyce‐Code (BCNF), and Cohen's Kappa (KAPPA) values ranged from −1 to +1, with a score of 1. AUC value of 0.5 represents the weakest predictive capability, equivalent to random prediction, whereas an AUC value of 1.0 indicates perfect model performance (Phillips et al. 2006; Wan et al. 2019).

Evaluation metrics revealed satisfactory performance across all four modelling approaches (GLM, GAM, Maxent, and RF). Based on AUC and TSS values, the Generalised Additive Model (GAM) provided the best fit for the Marbled Teal, while the Maximum Entropy (Maxent) model yielded the highest predictive performance for the Common Pochard. Model performance metrics for the best‐performing algorithms were as follows. For Marbled Teal, the GAM model achieved an AUC of 0.96 ± 0.02, TSS of 0.86 ± 0.05, specificity of 0.95, and sensitivity of 0.91. For Common Pochard, the Maxent model achieved an AUC of 0.95 ± 0.009, TSS of 0.78 ± 0.04, specificity of 0.85, and sensitivity of 0.92. The ensemble models, weighted by TSS and Boyce index, produced robust projections with consistently high performance across both species (see Table 1 for full algorithm comparison). These values indicate good‐to‐excellent predictive accuracy for both species.

**TABLE 1:** Comparative model performance for both species.

	Marbled Teal ( *Marmaronetta angustirostris* )					Common Pochard ( *Aythya ferina* )				
	AUC	TSS	Boyce	Specificity	Sensitivity	AUC	TSS	Boyce	Specificity	Sensitivity
GAM	**0.96 ± 0.02**	**0.86 ± 0.05**	0.53	0.95	0.91	0.94 ± 0.01	0.75 ± 0.06	0.78	0.88	0.87
GLM	0.86 ± 0.02	0.67 ± 0.09	0.44	0.89	0.78	0.88 ± 0.03	0.65 ± 0.05	0.71	0.78	0.87
Maxent	0.95 ± 0.02	0.83 ± 0.02	0.59	0.88	0.94	**0.95 ± 0.009**	**0.78 ± 0.04**	0.90	0.85	0.92
RF	0.95 ± 0.05	0.85 ± 0.06	0.83	0.94	0.90	0.94 ± 0.003	0.75 ± 0.05	0.95	0.95	0.80

*Note:* Bold values indicates *p* < 0.05.

**FIGURE 2:**
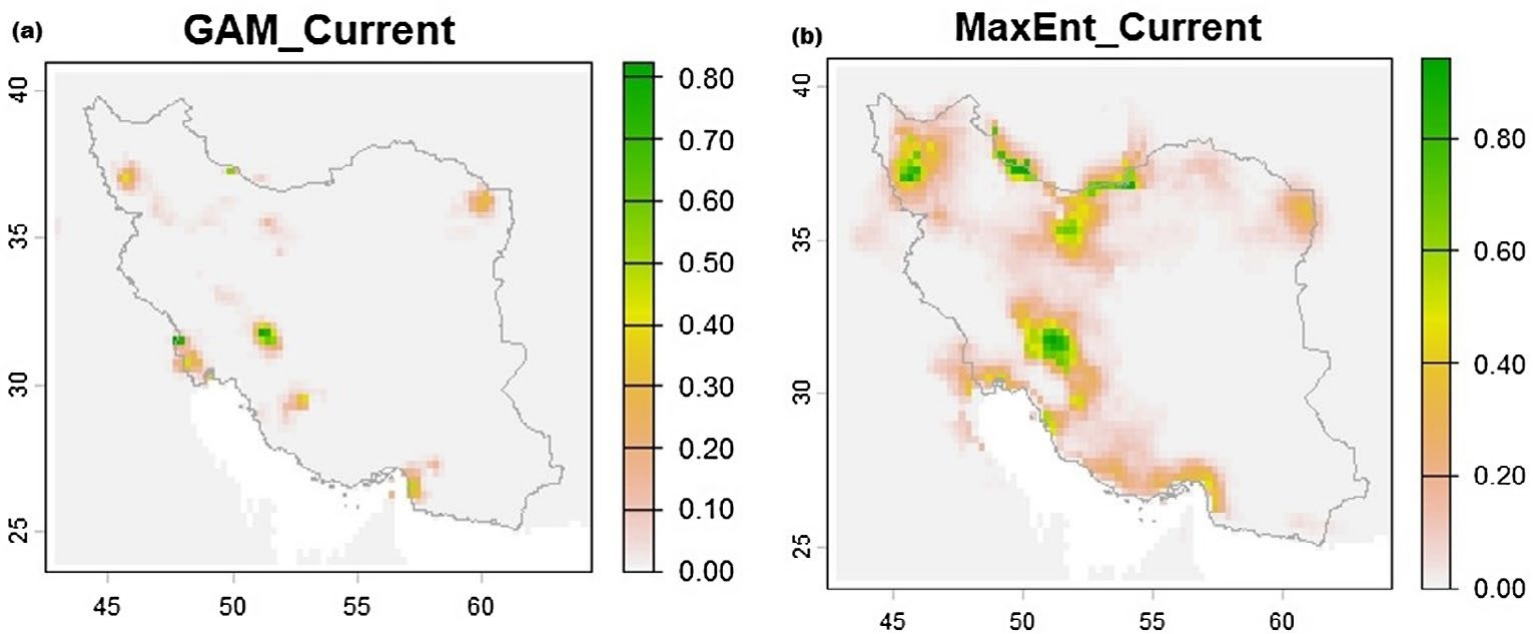
Current habitat suitability for (a) Marbled Teal (GAM model) and (b) Common Pochard (MaxEnt model). Warmer colours indicate higher suitability.

Regarding variable importance, the most influential environmental predictors for Marbled Teal were maximum temperature of the warmest month (Bio5, 76%) and land use/wetland (land 22: 99%). For Common Pochard, key predictors included land use/Sparse Forest and land use/orchards with percentage contributions of 99% and 53%, respectively (see Appendix 3 in Supporting Information for full variable importance rankings). The suitability maps indicate that Iran currently provides more suitable habitat for Common Pochard than for Marbled Teal, with suitable areas concentrated in montane regions and near the Caspian Sea coastline (Figure 2).

As a baseline for evaluating future habitat shifts, current habitat suitability was mapped for both species (Figure 2). The predicted distribution patterns revealed that suitable habitats are predominantly located within montane landscapes containing wetland habitats and in areas adjacent to the Caspian Sea coastline.

Species distribution models evaluated and predicted suitability based on species response to the environmental variables. This implies the importance of each environmental variable for the species in choosing an area as its home range. Therefore, the R program calculated the importance of each variable for each algorithm to show which environment has the most impact on suitability. To facilitate a more robust comparison of predictor contributions to habitat suitability projections, the relative importance of environmental variables was normalised for both the Marbled Teal and the Common Pochard (Appendix 3 in Supporting Information).

### 3.1 Altitudinal Analysis

**FIGURE 3:**
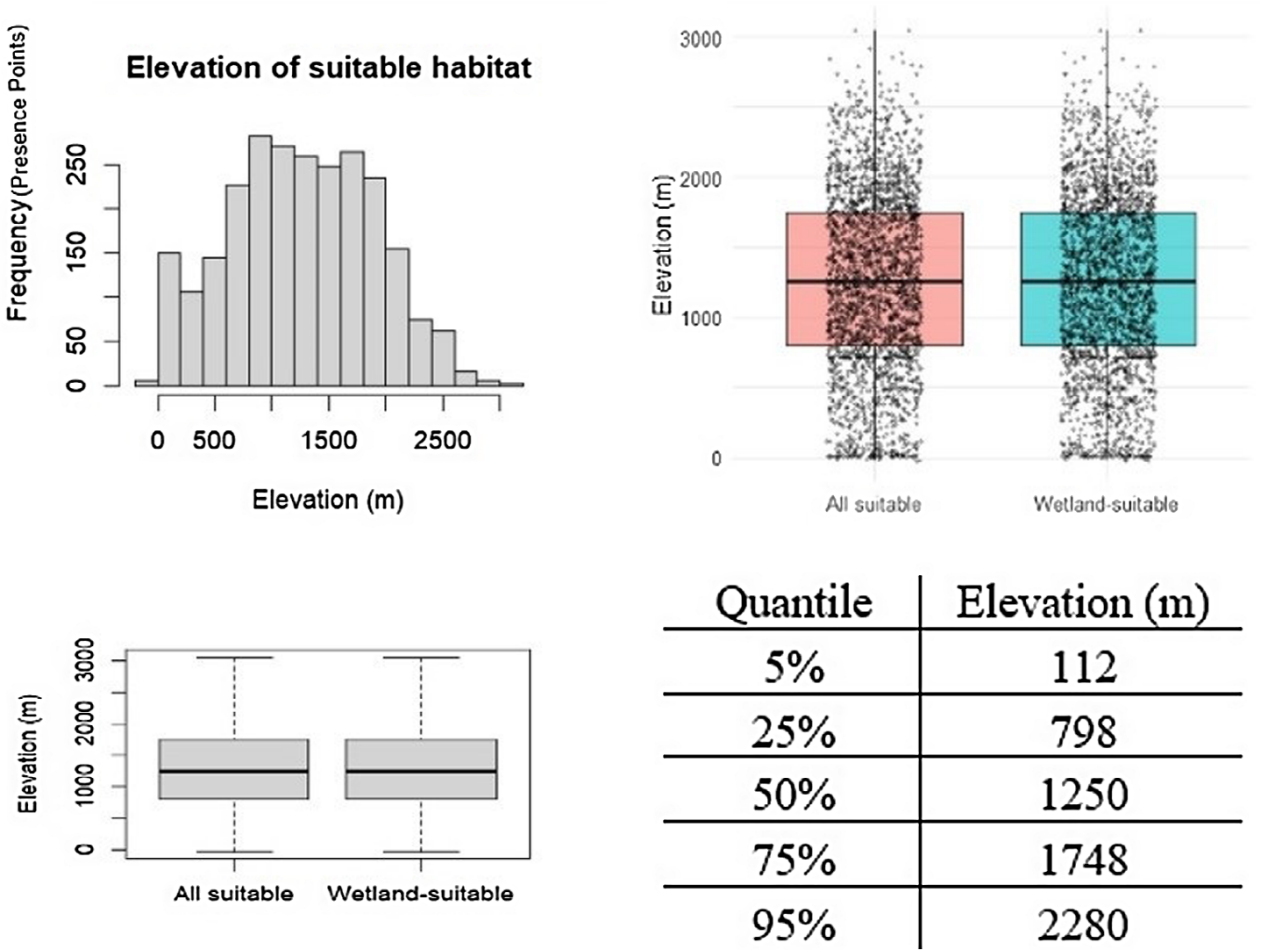
Elevation patterns were assessed using species occurrence data, spatial distribution analyses, histograms, and box plots. Wetland‐associated occurrences exhibited elevational distributions comparable to those of all suitable habitat records, suggesting that elevation exerts a limited influence on the spatial coincidence of suitable habitats and wetlands. Quantile analysis further demonstrated that over 75% of occurrence records were concentrated within moderate elevation ranges.

Although both species occur across a wide elevation gradient, the majority of records are concentrated at moderate elevations (1000–2000 m), and wetland‐associated presences follow the same pattern. Calculated median (1250 m), mean (1255 m), quartiles, and range for each category, which includes elevation‐frequency of presence, showed that the majority of occurrences (75%) of Common Pochard and Marbled Teal were located between 789 m (Q1) and 1748 m (Q3), with a median elevation of 1250 m (Figure 3).

### 3.2 Future Species Distribution Models

**FIGURE 4:**
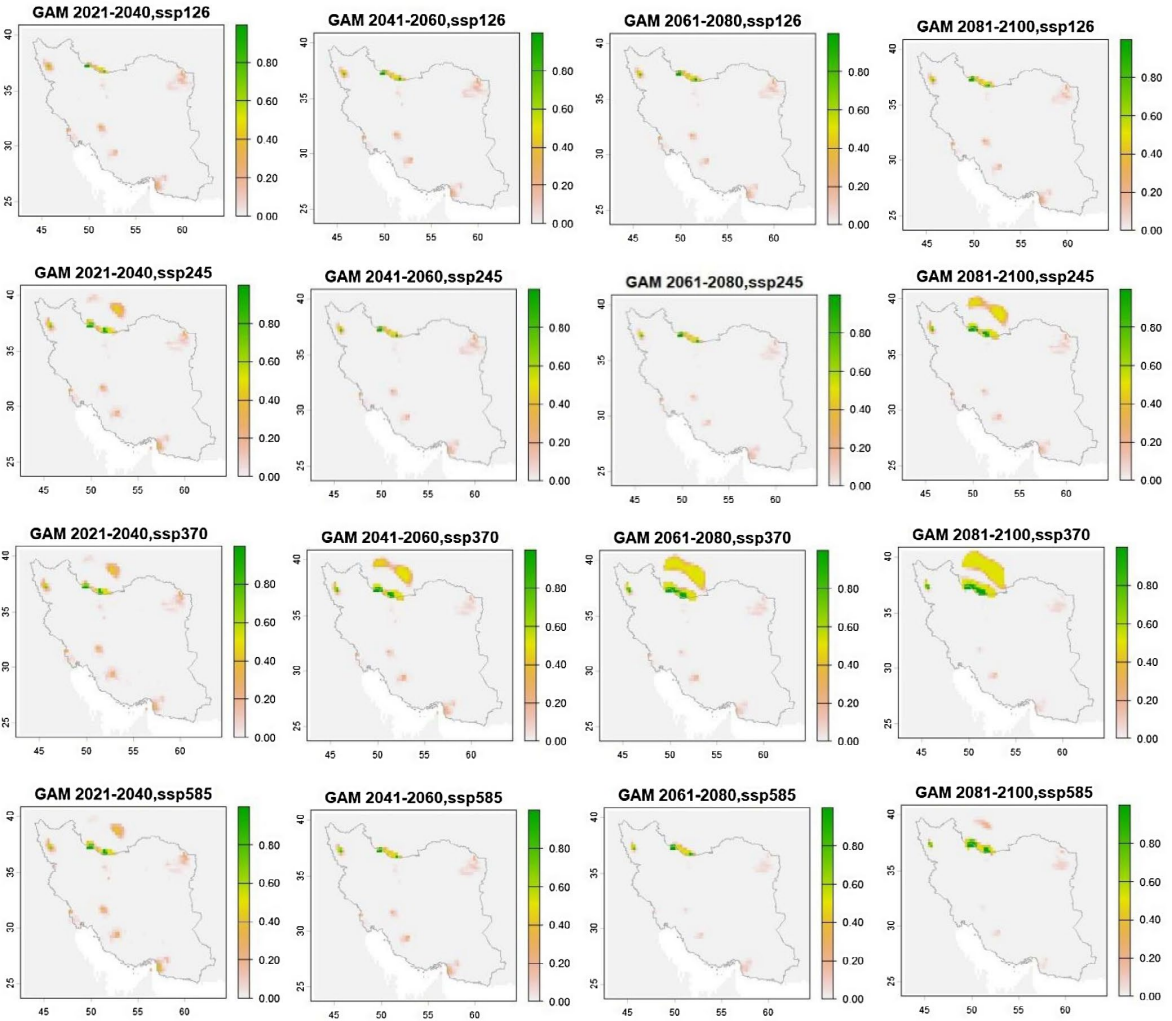
Predicted changes in Marbled Teal habitat suitability under climate change scenarios (2021–2100). Green indicates habitat gain, yellow stable suitable habitat, brown habitat loss, and white stable unsuitable habitat.

**FIGURE 5:**
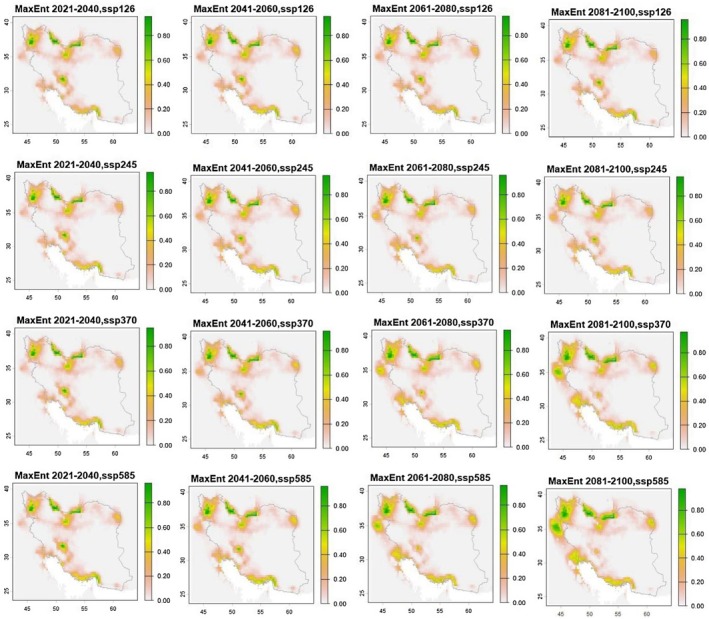
Projected changes in habitat suitability for the Common Pochard under future climate change scenarios (2021–2100). Colours represent habitat transition categories, with green indicating habitat gain, yellow denoting stable suitable habitat, brown representing habitat loss, and white indicating areas that remain unsuitable throughout the projection period.

Future habitat prediction was modelled under the SSP2‐RCP4.5 scenarios of climate‐land cover changes during 2021–2100 for Marbled Teal and Common Pochard in Iran. The suitability distribution shifting and extension area over time for both species is presented in Table 2 and in Figures 4 and 5.

**TABLE 2:** Probable distribution in the current and future habitat, with changing areas (Km^2^) for the species.

Marbled Teal (*Marmaronetta angustirostris*)	2021–2040	SSP	126	Marginal	35958	Common Pochard (*Aythya ferina*)	2021–2040	SSP	126	Marginal	318457
				Suitable	884					Suitable	18331
			245	Marginal	33982				245	Marginal	334988
				Suitable	3698					Suitable	17432
			370	Marginal	39053				370	Marginal	333693
				Suitable	3079					Suitable	18575
			585	Marginal	46531				585	Marginal	355241
				Suitable	3698					Suitable	18812
	2041–2060	SSP	126	Marginal	39070		2041–2060	SSP	126	Marginal	334880
				Suitable	1858					Suitable	17506
			245	Marginal	25205				245	Marginal	348424
				Suitable	2460					Suitable	16326
			370	Marginal	59943				370	Marginal	356968
				Suitable	5879					Suitable	16152
			585	Marginal	30174				585	Marginal	358858
				Suitable	1814					Suitable	18981
	2061–2080	SSP	126	Marginal	26196		2061–2080	SSP	126	Marginal	356708
				Suitable	2473					Suitable	15818
			245	Marginal	26331				245	Marginal	320355
				Suitable	2473					Suitable	16986
			370	Marginal	86249				370	Marginal	340030
				Suitable	8315					Suitable	22149
			585	Marginal	37272				585	Marginal	353689
				Suitable	2460					Suitable	22928
	2081–2100	SSP	126	Marginal	24513		2081–2100	SSP	126	Marginal	314251
				Suitable	2238					Suitable	16934
			245	Marginal	64960				245	Marginal	365266
				Suitable	5498					Suitable	15840
			370	Marginal	103484				370	Marginal	370441
				Suitable	10333					Suitable	26315
			585	Marginal	17472				585	Marginal	400383
				Suitable	5925					Suitable	37454
	Current			Marginal	43210		Current			Marginal	324122
				Suitable	2214					Suitable	15783

Under four future periods (2021–2040 to 2081–2100) and four SSP scenarios (126 to 585), both species show substantial habitat changes. For the Marbled Teal, current marginal habitat (43,210 km^2^) and suitable habitat (2214 km^2^) shift considerably: suitable habitat peaks at 10,333 km^2^ under SSP370 (2081–2100) but drops to 1814 km^2^ under SSP585 (2041–2060). For the Common Pochard, current marginal (324,122 km^2^) and suitable (15,783 km^2^) habitats expand under most scenarios, reaching maxima of 400,383 km^2^ and 37,454 km^2^, respectively, under SSP585 (2081–2100). These changes are largely attributable to the projected retreat of the Caspian Sea and expansion of adjacent wetlands. Under the SSP5–8.5 scenario (2081–2100), representing a climate change scenario used by the Intergovernmental Panel on Climate Change (IPCC). It combines a high‐growth, fossil fuel‐dependent socioeconomic pathway (SSP5) with extreme greenhouse gas emissions (RCP 8.5), resulting in a ‘worst‐case’ scenario where CO_2_ emissions triple by 2075; both species exhibited substantial increases in climatically suitable habitat. The Marbled Teal showed an expansion of up to 3711 km^2^, while the suitable habitat of the Common Pochard increased to 21,671 km^2^, corresponding to gains of 167.6% and 137.3%, respectively.

## 4 Discussion

Middle Eastern countries, especially Iran, are experiencing significant vulnerability to climate change (Namdar et al. 2021; Yousefi et al. 2019). Iran's unique geographical and geological features, including its towering Alborz and Zagros Mountain ranges, vast plateaus, and deep valleys, make it an ideal location for studying climate refugia.

The country's climate is notably diverse, featuring arid deserts in the central regions, temperate climates in the northern mountainous areas, and Mediterranean influences in the west (Yousefi et al. 2019). Historical studies in geomorphology, paleoecology, and palynology reveal that Iran has undergone substantial climate fluctuations during both the Pleistocene and Holocene epochs (Kehl 2009).

Identifying and understanding climate refugia in Iran is crucial, particularly in light of projections that indicate a significant temperature increase of 2.6°C and a concerning 35% reduction in precipitation (Mansouri Daneshvar et al. 2019). Moreover, various research findings suggest a potential temperature rise ranging from 1.12°C to 7.87°C by the year 2100 (Mansouri Daneshvar et al. 2019).

In light of ongoing climate change, we sought to forecast the future distribution of two duck species: the Common Pochard and the Marbled Teal. Among the 13 bioclimatic variables we examined, BIO1 and BIO5 had the most significant impact on the distribution of these species.

Our results indicate that, despite differences in their ecological niches and optimal model selection (GAM for Marbled Teal, Maxent for Common Pochard), both duck species show a similar response to future climate scenarios. They may expand into high‐elevation montane wetlands, particularly those above 1500 m. This trend indicates a shared resilience but also highlights a crucial reliance on the conservation of high‐altitude aquatic habitats, which are becoming essential climate refugia.

Our results identify both in situ refugia (areas currently suitable that remain suitable, shown in yellow in Figures 3 and 4) and potential ex‐situ refugia (areas of future gain, shown in green) in the Alborz and Zagros ranges. The SDMs show that the Caspian Sea coastline and adjacent wetlands, along with small, scattered areas overlapping with the wetlands in the Zagros Mountains, can act as climatic refuges over time under climate change scenarios. These results align with other studies conducted on Iranian species distributed in the same region (Rahimi et al. 2024; Vaissi and Mohammadi 2024). Hence, based on the SDMs' results in the face of shifting bioclimatic envelopes, in situ refugia can be used to guide habitat management.

The selection of GAM as the best model for the Marbled Teal reflects its strong non‐linear sensitivity to thermal variables, particularly annual mean temperature (Bio1) and maximum temperature of the warmest month (Bio5). This dependence on climatic factors explains its predicted altitudinal expansion: climate warming progressively makes previously colder montane habitats thermally more suitable, a phenomenon aligned with the ‘thermophilisation’ hypothesis of ecosystems. In contrast, for the Common Pochard, the MaxEnt model, which excels at integrating static environmental variables, was optimal, highlighting the predominant role of habitat availability and quality (e.g., land cover, proximity to large wetlands) over pure climatic variables. Its expansion into montane areas, therefore, appears to be driven more by the search for suitable habitats (wetlands) that escape lowland aridification, rather than by a direct thermal gradient. The Iranian context makes these findings particularly relevant. The country, characterised by a predominantly arid and semi‐arid climate, with projections indicating further temperature increases and a drastic reduction in precipitation, is already under significant water stress. Lowland wetlands, vital for waterfowl, are among the ecosystems most threatened by drought and human pressure. In this scenario, the mountain systems of the Alborz and Zagros, historically more humid and with greater persistence of water resources, take on a crucial role as microclimatic refuges. Our study provides modelling evidence supporting this function, identifying high‐elevation wetlands as areas of potential suitability gain for both species throughout the century.

However, this potential expansion is not without risks and uncertainties. Our models assume the future availability of adequate montane wetland habitats, ignoring potential dynamic changes in land use (e.g., increased agricultural or tourism pressure in high‐altitude areas) and complex hydrogeological processes that could alter water availability.

The conservation status of the two duck species studied in Iran is of concern. Efforts to monitor and protect their habitats, along with raising awareness about the importance of wetland conservation, are crucial for ensuring the continued survival of these species within Iran's borders. The MaxEnt and GAM models predicted that wetlands such as Hamun‐e‐Saberi, Hamun‐e‐Helmand, Gavkhouni, and Parishan would become unsuitable, while wetlands like Bujagh National Park, Anzali, Amirkelayeh, and Fereydoon Kenar would act as refugia.

To translate our projections into actionable conservation priorities, we assessed the overlap between future suitable habitats and Iran's existing protected area network. Under current conditions, 26% of suitable habitat for Common Pochard and 2% for Marbled Teal falls within nationally designated protected areas (e.g., National Parks, Wildlife Refuges, Protected Areas). Under the high‐emission scenario (SSP5‐8.5, 2081–2100), the proportion of suitable habitat inside protected areas is projected to change to 19% and 1%, respectively. This gap analysis reveals priority areas for targeted conservation interventions: (i) strengthening legal protection for existing montane wetlands that are projected to remain suitable (in situ refugia); (ii) establishing new protected areas or conservation easements in high‐elevation zones identified as future gain areas (ex‐situ refugia); and (iii) integrating climate‐resilient wetland networks into Iran's national biodiversity strategies and action plans.

The implications for conservation are clear and urgent. To protect the Common Pochard and Marbled Teal in Iran in the face of a changing climate, it is essential to (1) rigorously protect existing montane wetlands, formally recognising them as critical sites for the climate resilience of biodiversity; (2) furthermore, it is important to initiate ecological restoration programmes for degraded montane aquatic habitats, enhancing their water retention capacity and quality; (3) as well as to integrate future distribution scenarios into national species action plans, adapting monitoring and management strategies to areas identified as suitable in the long term. By implementing these measures, it could not only ensure that Iran's montane aquatic ecosystems act as a stronghold of current biodiversity but also become future arks of salvation for sensitive species like the Common Pochard and Marbled Teal.

## Author Contributions

**Abbas Naqibzadeh:** conceptualization (equal), formal analysis (equal), methodology (lead), resources (equal), software (lead), writing – original draft (equal), writing – review and editing (equal). **Ruhollah Asgari:** data curation (equal), methodology (equal), validation (equal), visualization (equal). **Cezary Mitrus:** validation (equal), visualization (equal), writing – review and editing (equal). **Attila Németh:** methodology (equal), project administration (equal), supervision (equal), validation (equal), visualization (equal), writing – original draft (equal), writing – review and editing (equal). **Salvatore Surdo:** methodology (equal), project administration (equal), supervision (equal), validation (equal), visualization (equal), writing – original draft (equal), writing – review and editing (equal).

## Funding

The authors have nothing to report.

## Conflicts of Interest

The authors declare no conflicts of interest.

## Supporting information


**Appendix 1.** Species presence.
**Appendix 2**. The environmental variables utilised to develop the Species Distribution Model (SDM).
**Appendix 3**. Normalised percentage importance of variables for Marbled Tail (GAM) and Common Pochard (MaxEnt).

## Data Availability

All environmental data used in this study are publicly available from WorldClim (https://www.worldclim.org) and GBIF (https://www.gbif.org). Species occurrence data include both publicly archived GBIF records and field survey data, which are included in the Supporting Information.
