# Supplementary material for: Prediction of the Potential Distribution of Common Pochard (Aythya ferina) and Marbled Teal (Marmaronetta angustirostris) in Iran Under Future Climate Scenarios
Source: Ecol Evol. 2026 Jul 6;16(7):e73971. doi: 10.1002/ece3.73971 (PMC13337331; doi:10.1002/ece3.73971)
Supplement: Supplementary file 1 — Appendix 1. Species presence. Appendix 2. The environmental variables utilised to develop the Species Distribution Model (SDM). Appendix 3. Normalised percentage importance of variables for Marbled Tail (GAM) and Common Pochard (MaxEnt). [file ECE3-16-e73971-s001.docx]

Appendix 1: Species presence

 Marbled Teal locality

| Latitude | Longitude | Latitude | Longitude | Latitude | Longitude | Latitude | Longitude |
| --- | --- | --- | --- | --- | --- | --- | --- |
| 36.09366 | 60.2103 | 27.0332 | 57.04971 | 36.27811 | 59.79494 | 31.83288 | 51.12364 |
| 36.27811 | 59.79494 | 31.26294 | 47.88523 | 31.23816 | 47.78487 | 31.83268 | 51.12574 |
| 31.26294 | 47.88523 | 31.26294 | 47.88523 | 36.99104 | 45.77265 | 31.83359 | 51.12354 |
| 31.38271 | 47.77707 | 31.83535 | 51.10093 | 29.66575 | 53.20932 | 31.83309 | 51.12097 |
| 31.26294 | 47.88523 | 36.99104 | 45.77265 | 30.64017 | 48.14312 | 31.83318 | 51.12237 |
| 31.47812 | 47.85855 | 37.45953 | 49.93863 | 37.03826 | 45.59258 | 31.83249 | 51.12481 |
| 36.09366 | 60.2103 | 26.60923 | 57.16077 | 31.26294 | 47.88523 | 31.83259 | 51.12783 |
| 36.27811 | 59.79494 | 36.99104 | 45.77265 | 31.83535 | 51.10093 | 31.83189 | 51.12619 |
| 35.46378 | 51.52049 | 31.47812 | 47.85855 | 29.66934 | 53.17144 | 31.83528 | 51.12473 |
| 31.38271 | 47.77707 | 31.26294 | 47.88523 | 31.30076 | 47.73746 | 31.83389 | 51.12319 |
| 36.27811 | 59.79494 | 31.26294 | 47.88523 | 34.73002 | 49.31347 | 31.83278 | 51.12364 |
| 29.44238 | 52.76521 | 31.26294 | 47.88523 | 31.26294 | 47.88523 | 31.83319 | 51.12691 |
| 31.38271 | 47.77707 | 31.26294 | 47.88523 | 37.03826 | 45.59258 | 31.83329 | 51.11689 |
| 31.26294 | 47.88523 | 37.03826 | 45.59258 | 37.03826 | 45.59258 | 31.83698 | 51.12359 |
| 31.47812 | 47.85855 | 31.26294 | 47.88523 | 31.36876 | 47.78946 | 31.83458 | 51.12344 |
| 31.26294 | 47.88523 | 37.05933 | 45.46495 | 37.03826 | 45.59258 | 31.83428 | 51.12516 |
| 36.09366 | 60.2103 | 31.26294 | 47.88523 | 31.26294 | 47.88523 | 31.83249 | 51.12539 |
| 36.09366 | 60.2103 | 36.99104 | 45.77265 | 28.93514 | 50.90583 | 31.83239 | 51.1262 |
| 31.26294 | 47.88523 | 31.26294 | 47.88523 | 31.83535 | 51.10093 | 31.83428 | 51.12588 |
| 36.20303 | 59.63225 | 26.35369 | 57.34411 | 31.83535 | 51.10093 | 31.83228 | 51.1276 |
| 37.45953 | 49.93863 | 36.99104 | 45.77265 | 31.83535 | 51.10093 | 31.26294 | 47.88523 |
| 26.35369 | 57.34411 | 36.99104 | 45.77265 | 31.83535 | 51.10093 | 36.99104 | 45.77265 |
| 26.35369 | 57.34411 | 36.99104 | 45.77265 | 31.23816 | 47.78487 | 27.0332 | 57.04971 |
| 36.09366 | 60.2103 | 36.99104 | 45.77265 | 31.38271 | 47.77707 | 26.35552 | 57.34916 |
| 31.26294 | 47.88523 | 31.26294 | 47.88523 | 29.66934 | 53.17144 | 37.45953 | 49.93863 |
| 31.26294 | 47.88523 | 36.09366 | 60.2103 | 34.73002 | 49.31347 | 31.83199 | 51.12724 |
| 27.0332 | 57.04971 | 36.09366 | 60.2103 | 31.36876 | 47.78946 | 31.83289 | 51.12772 |
| 31.2451 | 47.78486 | 31.26294 | 47.88523 | 31.83535 | 51.10093 | 31.83249 | 51.12527 |
| 26.35369 | 57.34411 | 27.0332 | 57.04971 | 36.00492 | 60.39036 | 31.83189 | 51.12433 |
| 26.60923 | 57.16077 | 37.03079 | 45.44014 | 27.16219 | 56.8218 | 31.83179 | 51.12526 |
| 31.47812 | 47.85855 | 36.27811 | 59.79494 | 31.83478 | 51.12507 | 36.27811 | 59.79494 |
| 26.35369 | 57.34411 | 37.03826 | 45.59258 | 31.83477 | 51.12647 | 27.0332 | 57.04971 |
| 26.35369 | 57.34411 | 31.47812 | 47.85855 | 31.83388 | 51.12622 | 26.60923 | 57.16077 |
| 26.35552 | 57.34916 | 36.99104 | 45.77265 | 31.83278 | 51.12609 | 31.26294 | 47.88523 |
| 26.35552 | 57.34916 | 36.99104 | 45.77265 | 31.83309 | 51.12749 |  |  |

Common Pochard Locality

| Latitude | Longitude | Latitude | Longitude | Latitude | Longitude | Latitude | Longitude |
| --- | --- | --- | --- | --- | --- | --- | --- |
| 37.46508 | 49.33102 | 37.39361 | 49.45326 | 38.1856 | 46.90996 | 31.83228 | 51.12771 |
| 36.9982 | 45.43478 | 36.90797 | 54.01909 | 36.9026 | 54.01914 | 31.83278 | 51.12632 |
| 37.45688 | 49.92462 | 36.99104 | 45.77265 | 31.66184 | 48.66058 | 31.83259 | 51.12481 |
| 29.5893 | 52.50374 | 30.64017 | 48.14312 | 36.89717 | 54.04276 | 31.83309 | 51.12703 |
| 37.20509 | 50.01425 | 34.84185 | 59.9572 | 37.46067 | 49.92982 | 31.83249 | 51.11036 |
| 37.18457 | 50.16084 | 31.30078 | 47.7374 | 37.46859 | 49.93966 | 31.832 | 51.10698 |
| 35.76201 | 52.56026 | 36.8995 | 53.9857 | 36.6032 | 60.26064 | 31.8315 | 51.11129 |
| 35.94163 | 50.42997 | 31.26294 | 47.88523 | 36.99577 | 45.56631 | 31.8307 | 51.1042 |
| 29.58941 | 52.50214 | 37.91692 | 46.70039 | 37.03908 | 45.59586 | 31.83239 | 51.11315 |
| 36.09366 | 60.2103 | 36.67011 | 52.46607 | 38.36976 | 48.84964 | 31.83249 | 51.11514 |
| 37.42836 | 54.639 | 37.50153 | 49.35373 | 28.90569 | 50.99125 | 31.83041 | 51.10757 |
| 35.82221 | 55.13334 | 31.9408 | 50.88595 | 26.35655 | 57.34772 | 31.83318 | 51.12399 |
| 28.93514 | 50.90583 | 36.87181 | 53.50244 | 27.53752 | 56.88867 | 31.83199 | 51.12363 |
| 34.82265 | 48.61755 | 38.86771 | 45.65294 | 38.86261 | 45.65237 | 31.83268 | 51.12562 |
| 35.3382 | 51.34372 | 36.71594 | 52.97489 | 27.55198 | 56.85061 | 31.83276 | 51.12749 |
| 36.91897 | 54.02048 | 27.17306 | 53.17686 | 31.83817 | 51.12314 | 31.83528 | 51.12484 |
| 29.12504 | 51.59399 | 38.86495 | 45.65635 | 31.83442 | 51.12873 | 31.83658 | 51.12405 |
| 36.99989 | 45.56839 | 36.83614 | 53.56068 | 29.57506 | 52.53714 | 31.83698 | 51.12276 |
| 35.46378 | 51.52049 | 37.474 | 49.34511 | 37.18139 | 50.16346 | 31.83879 | 51.12305 |
| 37.03079 | 45.44014 | 27.7971 | 52.56966 | 37.20439 | 50.01139 | 31.8406 | 51.11671 |
| 30.27299 | 49.98166 | 37.49233 | 49.31909 | 37.20619 | 50.01129 | 37.20628 | 50.01015 |
| 29.44238 | 52.76521 | 38.19661 | 46.87183 | 37.20704 | 50.01004 | 37.18284 | 50.16286 |
| 35.94935 | 50.46259 | 38.37645 | 48.84705 | 31.83441 | 51.1154 | 36.5561 | 52.63598 |
| 37.39335 | 49.45805 | 36.78711 | 53.90542 | 31.83382 | 51.1119 | 37.20623 | 50.01511 |
| 36.06967 | 60.22302 | 31.38271 | 47.77707 | 31.84333 | 51.11309 | 37.20701 | 50.01081 |
| 36.08648 | 60.28757 | 35.3363 | 51.34382 | 31.84182 | 51.11425 | 36.99851 | 45.57503 |
| 37.03826 | 45.59258 | 37.37188 | 54.58237 | 31.83949 | 51.11846 | 37.04149 | 45.5243 |
| 35.74628 | 51.20783 | 35.46266 | 51.51835 | 31.84425 | 51.11027 | 37.03684 | 45.49591 |
| 36.9922 | 45.43625 | 35.3087 | 51.4741 | 31.84338 | 51.11144 | 37.03588 | 45.48853 |
| 30.43306 | 52.13193 | 27.0507 | 54.95543 | 27.30117 | 56.05192 | 37.04712 | 45.47823 |
| 35.50057 | 51.49043 | 26.87846 | 54.77211 | 35.00042 | 51.34412 | 35.30363 | 51.47376 |
| 35.76703 | 52.55927 | 36.78814 | 53.94211 | 35.36792 | 51.28869 | 35.30804 | 51.46475 |

Appendix 2: The environmental variables utilised to develop the Species Distribution Model (SDM)

| **Variables** | **Environmental Variables Description** | **Approach** |
| --- | --- | --- |
| Bio1 | Annual Mean Temperature | Ecological/Statistical |
| Bio2 | Mean Diurnal Range (Mean of monthly (max temp - min temp)) | Ecological |
| Bio3 | Isothermality (BIO2/BIO7) (×100) | Ecological |
| Bio5 | Max Temperature of Warmest Month | Ecological/Statistical |
| Bio6 | Min Temperature of Coldest Month | Ecological |
| Bio7 | Temperature Annual Range (BIO5-BIO6) | Ecological |
| Bio8 | Mean Temperature of Wettest Quarter | Ecological |
| Bio9 | Mean Temperature of Driest Quarter | Ecological |
| Bio12 | Annual Precipitation | Ecological/Statistical |
| Bio13 | Precipitation of Wettest Month | Ecological |
| Bio15 | Precipitation Seasonality (Coefficient of Variation) | Ecological |
| Bio17 | Precipitation of the Driest Quarter | Ecological/Statistical |
| Bio18 | Precipitation of Warmest Quarter | Ecological |
| Eco56 | Badghyz and Karabil semi-desert | Ecological/Statistical |
| Eco652 | South Iran Nubo-Sindian desert and semi-desert | Ecological/Statistical |
| Eco742 | Tigris-Euphrates alluvial salt marsh | Ecological/Statistical |
| Land3 | Orchards | Ecological/Statistical |
| Land8 | Dense pasture | Ecological/Statistical |
| Land10 | Island | Ecological/Statistical |
| Land12 | Mangrove forest | Ecological/Statistical |
| Land13 | Marginal land, environmental, and/or socioeconomic constraints | Ecological/Statistical |
| Land14 | Moderately dense forest (MDF), [canopy cover](https://www.google.com/search?q=canopy+cover&client=firefox-b-d&hs=GFeU&sca_esv=55a4e0c02f0bf2c1&biw=1366&bih=635&sxsrf=ANbL-n5skrtusB4qSj5SkM67BwzCHARnYQ%3A1770564636299&ei=HKyIadjfEbW0i-gPx6WqiAU&oq=moderate+density+forest&gs_lp=Egxnd3Mtd2l6LXNlcnAiF21vZGVyYXRlIGRlbnNpdHkgZm9yZXN0KgIIADIGEAAYFhgeMgYQABgWGB4yBhAAGBYYHjIGEAAYFhgeMgYQABgWGB4yCxAAGIAEGIYDGIoFMggQABiABBiiBDIFEAAY7wUyCBAAGIAEGKIEMggQABiiBBiJBUiXUFCKBlj5O3AFeACQAQCYAaQBoAHZFaoBBTEwLjE1uAEByAEA-AEBmAIeoALSGMICDhAAGIAEGLADGIYDGIoFwgIIEAAYsAMY7wXCAgsQABiABBiwAxiiBMICBBAjGCfCAgoQIxiABBgnGIoFwgIKEC4YgAQYJxiKBcICCxAAGIAEGJECGIoFwgIFEAAYgATCAgUQLhiABMICCxAuGIAEGNEDGMcBwgIKECMY8AUYJxjJAsICCxAuGIAEGJECGIoFwgIKEAAYgAQYQxiKBcICChAuGIAEGEMYigXCAgoQABiABBgUGIcCwgIIEAAYFhgKGB6YAwCIBgGQBgeSBwQ3LjIzoAeq3QGyBwQyLjIzuAeNGMIHCDItNi4yMy4xyAegAoAIAA&sclient=gws-wiz-serp&ved=2ahUKEwj8xeu-m8qSAxWZ9QIHHXuSIg4QgK4QegQIARAB) 40% - 70% | Ecological/Statistical |
| Land21 | Sparse forest | Ecological/Statistical |
| Land22 | Wetlands | Ecological/Statistical |
| Land23 | Woodland | Ecological/Statistical |
| Soil | Soil moisture | Ecological/Statistical |

Appendix 3. Normalised percentage importance of variables for Marbled Tail (GAM) and Common Pochard (MaxEnt)
